# Application of artificial intelligence in hepatology

**DOI:** 10.3389/fdgth.2026.1851723

**Published:** 2026-09-16

**Authors:** Zekai Hu, Yajie Lin, Hainv Gao, Leyao Shuai, Zhaowei Tong, Chenqi Nie, Zhe Yang, Junsheng Zhao

**Affiliations:** 1 Bioinformatic Center, Key Laboratory of Artificial Organs and Computational Medicine of Zhejiang Province, Shulan (Hangzhou) Hospital, Shulan International Medical College, Zhejiang Shuren University, Hangzhou, China; 2 Department of Infection, Key Laboratory of Artificial Organs and Computational Medicine of Zhejiang Province, Shulan (Hangzhou) Hospital, Shulan International Medical College, Zhejiang Shuren University, Hangzhou, China; 3 Department of Infectious Diseases, Huzhou Central Hospital, Affiliated Central Hospital, Huzhou University, Huzhou, Zhejiang, China; 4 Huzhou Key Laboratory of Precision Medicine Research and Translation for Infectious Diseases, Affiliated Central Hospital, Huzhou University, Huzhou, Zhejiang, China; 5 Department of Hepatobiliary and Pancreatic Surgery, Key Laboratory of Artificial Organs and Computational Medicine in Zhejiang Province, Shulan (Hangzhou) Hospital, Shulan International Medical College, Zhejiang Shuren University, Hangzhou, China; 6 Department of Hepatobiliary and Pancreatic Surgery, Shulan (Boao) Hospital, Boao, China; 7 State Key Laboratory for Diagnosis and Treatment of Infectious Diseases, National Clinical Research Center for Infectious Diseases, National Medical Center for Infectious Diseases, Collaborative Innovation Center for Diagnosis and Treatment of Infectious Diseases, The First Affiliated Hospital, Zhejiang University School of Medicine, Hangzhou, China

**Keywords:** artificial intelligence, clinical implementation, diagnosis, liver disease, treatment planning

## Abstract

Artificial intelligence (AI) is being applied across diagnostic and therapeutic workflows in hepatology. This narrative review summarizes recent advances in AI for liver disease. In medical imaging and digital pathology, computer vision enables automated quantitative analysis of ultrasound, CT, MRI, and histologic images, with the aim of improving the consistency of lesion detection, disease staging, and prognostic assessment. In biomarker research, machine learning can analyze high-dimensional liquid-biopsy and multi-omics data to develop diagnostic and prognostic models; some have outperformed conventional markers in their study cohorts. Electronic health records (EHRs) and large language models (LLMs) are also being investigated for clinical decision support and personalized management. However, most reported evidence remains retrospective, and clinical adoption is limited by data heterogeneity, poor interpretability, uncertain generalizability, and regulatory requirements. Progress will require standardized datasets, external and prospective validation, clinically relevant endpoints, and human-centered implementation before gains in model performance can be translated into better patient outcomes.

## 1 Introduction

Liver disease represents a major and growing global health challenge and contributes substantially to morbidity and mortality worldwide (1–5). Cirrhosis and liver cancer collectively account for more than 2 million deaths each year and rank among the leading causes of death globally (6). The epidemiology of liver disease is diverse, and its predominant etiologies are changing. Historically, hepatitis B virus (HBV) and hepatitis C virus (HCV) have been major causes of chronic liver injury, cirrhosis, and hepatocellular carcinoma (HCC) (5, 7). Widespread HBV vaccination and highly effective direct-acting antiviral treatment for HCV have begun to change this pattern and may reduce virus-related end-stage liver complications (7). This progress is being offset by the rising prevalence of metabolic dysfunction-associated steatotic liver disease (MASLD) (8, 9). MASLD is closely associated with the global epidemics of obesity and type 2 diabetes and is now the most prevalent chronic liver disease worldwide (10–12). It affects an estimated 38% of adults and 7% to 14% of children and adolescents, and its prevalence among adults is projected to exceed 55% by 2040 (12, 13). The prevalence of metabolic dysfunction-associated steatohepatitis (MASH) is projected to increase by 20% to 35% by 2030 (12). Alcohol-related liver disease also continues to contribute substantially to the disease burden (14–16). Autoimmune liver diseases, including autoimmune hepatitis and primary biliary cholangitis, as well as genetic disorders and toxicant-induced liver injury, add further complexity.

Managing the global burden of liver disease is difficult because its causes are complex, multifactorial, and often synergistic (17, 18). Sedentary behavior, diets rich in ultra-processed foods, fructose, and saturated fats, and chronic psychosocial stress contribute to the development and progression of MASLD (19). This metabolic burden often coexists with other risk factors. For example, patients with MASLD may have resolved HBV infection, consume alcohol at harmful levels, and have obesity, a combination that further increases the risk of fibrosis and HCC. This convergence of etiologies complicates prevention. One report estimated that more than 60% of liver cancer cases worldwide could be prevented by addressing viral hepatitis, alcohol use, and MASLD, and projected that the global number of new cases could double by 2050 without effective intervention (20). Liver disease often progresses without symptoms, leading to diagnosis at stages when treatment options are more limited, costly, and less effective. Current diagnostic pathways rely on nonspecific serum biomarkers, imaging, and liver biopsy, historically regarded as the reference standard, but each approach has limitations (21). Liver biopsy is invasive, is susceptible to sampling error, and carries a risk of complications, which limits its suitability for population screening and longitudinal monitoring (22). These limitations, together with the increasingly complex epidemiology of liver disease, create a need for better approaches to diagnosis and management.

Growth in computational capacity, including graphics processing units and tensor processing units, together with advances in algorithms, has expanded the use of AI in medical research and clinical practice. AI applications in healthcare can be grouped into four interconnected domains. First, computer vision (CV) uses methods such as convolutional neural networks (CNNs), generative adversarial networks (GANs), and vision transformers (ViTs) to analyze images (23, 24). Second, machine learning (ML) includes support vector machines (SVMs), random forests, and deep neural networks (DNNs); self-supervised learning can reduce annotation requirements. Third, natural language processing (NLP) uses Transformer-based LLMs, while recurrent neural networks (RNNs) and long short-term memory networks remain in use for sequential data. Fourth, multimodal AI combines image, text, and numerical data through early, intermediate, or late fusion. These approaches have enabled models such as Delphi-2M, which was reported to predict the risk of more than 1,000 diseases up to 20 years in advance from medical records and lifestyle data (25). Unified-IO 2 is a multimodal model that integrates several data sources to provide a broader representation of disease-related information (26).

Hepatology may benefit substantially from AI because it encompasses diverse conditions, including MASLD, MASH, and HCC, relies heavily on multimodal data, and requires earlier detection and more individualized management (Figure 1) (27). AI methods may identify features that are difficult to detect by conventional analysis, support dynamic risk stratification, and inform treatment selection. This narrative review examines AI applications in liver radiology for tumor detection and fibrosis assessment, digital pathology for fibrosis and MASH staging, and liquid-biopsy and multi-omics analysis for early detection and longitudinal monitoring. It also considers the use of NLP and LLMs to analyze EHR data and the use of multimodal models to combine imaging, text, and numerical data (Figure 2). These applications are evaluated in relation to early diagnosis, risk stratification, individualized treatment, and workflow efficiency, including liver transplantation. The review also examines limitations involving data standardization, model generalizability, clinical validation, and workflow integration. Its aim is to identify requirements for the responsible translation of AI from research into routine hepatology practice.

## 2 Methods

### 2.1 Review approach

This article is a narrative review, PubMed/MEDLINE was searched and the reference set was updated through 23 March 2026 using combinations of hepatology terms (liver disease, cirrhosis, hepatocellular carcinoma, MASLD, MASH, and liver transplantation) with AI terms (artificial intelligence, machine learning, deep learning, computer vision, radiomics, digital pathology, large language model, natural language processing, electronic health record, and multi-omics). English-language human studies that addressed a clinically relevant hepatology task and reported sufficient methodological or performance information were prioritized, particularly multicenter, external, or prospective validation studies and official regulatory documents. Duplicates, non-hepatology applications, and purely technical reports without a defined clinical task were excluded. Reference lists of eligible articles and relevant reviews were hand-searched.

### 2.2 Evidence appraisal framework

To separate analytical performance from clinical readiness, representative studies were appraised using five descriptive translational maturity levels (Tables 1–3): level 1, model development with internal validation; level 2, independent external validation; level 3, prospective or multicenter clinical validation; level 4, an authorized device or implemented workflow with defined lifecycle oversight; and level 5, demonstrated benefit to patients or health systems. Study design, setting, validation, endpoint, and regulatory or implementation status were considered separately. Diagnostic accuracy, segmentation overlap, and workflow measures were treated as surrogate endpoints, whereas mortality, decompensation, treatment allocation, and health-system outcomes were treated as patient-relevant or system-relevant endpoints. These levels summarize translational maturity and are not a formal risk-of-bias instrument.

**Table 1:** Summary and evidence appraisal of AI-based medical imaging models in liver disease.

Focus	Imaging modality	Dataset, design, and validation	Performance, endpoint, and translational status	Algorithm	OCEBM 2011 level	Reference
Classify six types of focal liver lesions	US	3,725 patients; US videos, immunohistochemical biomarkers, and clinical information; retrospective development across 52 hospitals; three prospective external test cohorts	Accuracy 0.85–0.86 across three external test cohorts; six-class diagnostic accuracy, surrogate endpoint; translational level 3, research model	ResNet, LSTM, MLP	3 (Dx)	Ding et al. (46)
Differentiate indeterminate liver nodules	US	244 indeterminate hepatic nodules in 224 patients	sensitivity of 56.3% and specificity of 93.9%	Classification Tree	3 (Dx)	Hoopes et al. (49)
Screen for advanced liver fibrosis	US	3,067 patients; liver US images; retrospective study across three facilities; internal and independent external testing	Specificity 94%; positive predictive value 73%; unnecessary referrals reduced by 35%; biopsy-referenced diagnosis and simulated referral reduction, surrogate/workflow endpoints; translational level 2, research model	DCNN	3 (Dx)	Chen et al. (41)
Diagnosis of echinococcosis	US	9,631 liver US images of 6,784 patients	The AUCs for differentiating echinococcosis from other liver lesions were 0.984, for differentiating alveolar echinococcosis from cystic echinococcosis were 0.986, and for assessing cystic echinococcosis activity were 0.974	DCNN	3 (Dx)	Yang et al. (47)
Differentiate HCC ≤3 cm from non-HCC lesions	US	US images of 1,058 liver lesions in 1,052 patients	The AUC of model URC using XGBoost was 0.934	MLP, XGBoost	3 (Dx)	Du et al. (48)
Quantify liver stiffness and fat fraction	US	78 enrolled and 70 analyzed participants; prospective single-center clinical-accuracy study; biopsy comparison and FDA 510(k) review	AUC 0.861 for significant fibrosis, 0.791 for advanced fibrosis, and 0.846 for moderate steatosis by VDFF; biopsy-referenced quantitative endpoints; translational level 4, FDA-cleared for a defined intended use; patient benefit not established	Quantitative ultrasound device	3 (Dx)	Sheikh et al. (51); FDA (52)
Opportunistically screen for cirrhosis and steatotic liver disease	Echocardiography	1,596,640 echocardiography videos from 24,276 patients; retrospective observational study at two centers; external cohort of 106 patients	External AUROC 0.830 for cirrhosis and 0.769 for steatotic liver disease; surrogate screening endpoints; translational level 2, research model	Deep neural network	3 (Dx)	Sahashi et al. (55)
Diagnose hepatocellular carcinoma	MRI	1,023 focal-lesion cases from 839 patients; retrospective multicenter diagnostic study; independent external test set	AUC 0.98 in training and 0.97 in the independent external test set; malignancy classification and radiologist-assistance metrics, surrogate endpoints; translational level 2, research model	ResNet	3 (Dx)	Li et al. (35)
Predict the microvascular invasion and recurrence-free survival of HCC patients	MRI	725 patients	The AUC value predicted by microvascular invasion was 0.918, and the c-index of recurrence-free survival was 0.763	Transformer	3 (Px)	Wang et al. (64)
Lesions were segmented and focal liver lesions were classified	MRI	2,131 individuals with focal liver lesions; retrospective development with multicenter external and prospective test cohorts	Mean Dice coefficients 0.98 (liver) and 0.96 (tumor); feature and lesion classification accuracies 93% and 97%; segmentation and lesion classification, surrogate/workflow endpoints; translational level 3, research model	CNN	3 (Dx)	Shen et al. (57)
Classify benign, primary malignant, and metastatic liver lesions	MRI	50,418 MRI images of 1,959 patients with liver tumors from three centers	The AUC of benign, primary malignant or metastatic liver lesions classification was 0.91, 0.873 and 0.876, respectively	CNN	3 (Dx)	Zhen et al. (58)
Cardiovascular risk assessment	MRI	44,672 liver MRIs	For predicting major adverse cardiac events, the AUC was 0.70	Transformer	3 (Px)	Veldhuizen et al. (63)
Automatic liver and spleen segmentation and liver fibrosis classification and decision model were performed	CT	CT data of 994 patients	The AUC was 0.92	ResNet, LSTM	3 (Dx)	Zheng et al. (65)
Classify focal liver lesions	CT	CT images of 4,039 patients	The accuracy of LiLNet for benign and malignant tumors was 94.7%, and the AUC was 97.2%. For HCC, ICC and MET, the accuracy was 88.7% and the AUC was 95.6%. For FNH, HEM and cysts, the accuracy was 88.6% and the AUC was 95.9%.	DCNN	3 (Dx)	Wei et al. (68)
Predict HCC risk in chronic hepatitis B	CT	CT images from 8,468 patients with chronic hepatitis B; retrospective multicenter cohort; independent external validation	C-index 0.91 in internal validation and 0.89 in independent external validation; 10-year HCC incidence, patient-relevant prognostic endpoint; translational level 2, research model	GBM	3 (Px)	Shin et al. (66)
Automated liver tumor detection and depiction	CT	CT data of 1,598 CT scans from 1,306 cancer patients.	The detection accuracy for patients was 99.65%, the detection accuracy for lesion levels was 81.72%, and DICE=0.760	3D U-Net	3 (Dx)	Balaguer-Montero et al. (69)
CT was used to diagnose HCC.	CT	CT data of 4,305 CT scans from 2,832 patients	The AUC was 0.919, and the negative predictive value was 0.966.	ST3DCN	3 (Dx)	Yu et al. (70)
Classify focal liver lesions and triage low-risk cases	CT	11,385 retrospective patients and 1,225 prospective patients from 18 hospitals; multicenter internal and prospective testing	AUC 0.982; diagnosis and triage, surrogate/workflow endpoints; translational level 3, research model	RPN,R-CNN, Transformer	3 (Dx)	Ying et al. (67)
Validate accelerated PET acquisition	PET	120 patients with PET/CT	The sensitivity and specificity at the lesion level were 95.45% and 98.41%, respectively	U-Net	3 (Dx)	Long et al. (72)

DCNN, deep convolutional neural network; Dx, diagnosis/monitoring question; GBM, gradient boosting machine; LSTM, long short-term memory; MLP, multilayer perceptron; OCEBM, Oxford Centre for Evidence-Based Medicine; Px, prognosis question; R-CNN, region-based convolutional neural network; ResNet, residual network; RPN, region proposal network; ST3DCN, spatial-temporal three-dimensional convolutional network; VDFF, velacur-derived fat fraction. For representative applications, study design, setting, validation, endpoint type, and evidence status are reported in the dataset and performance columns. Translational maturity levels are descriptive and defined in Section 1; no representative application demonstrated level 5 patient or health-system benefit. The OCEBM 2011 level was assigned by the primary clinical question and reported study design (28). Diagnostic-accuracy studies described as screening were appraised in the diagnosis/monitoring domain because the OCEBM screening hierarchy concerns whether early detection improves outcomes. Missing information on consecutive enrollment, blinding, and consistent reference-standard application was graded conservatively. OCEBM levels do not replace a study-specific risk-of-bias assessment.

**Table 2:** Summary and evidence appraisal of deep learning models based on pathologic images in liver disease.

Focus	Dataset, design, and validation	Performance, endpoint, and translational status	Algorithm	OCEBM 2011 level	Reference
Automatically segment portal-tract regions in H&E-stained whole-slide images	223 cases of WSI from multi-institutional	Dice coefficient 0.92; intersection-over-union score 0.86	Transformer	3 (Dx)	Cazzaniga et al. (88)
Support standardized MASH histologic scoring	12,458 cases of WSI	Overlay success rate for ballooning true-positive regions 0.87; reported true-positive and false-positive region success measures for other features approximately 0.92–1.00	CNN, GNN	3 (Dx)	Pulaski et al. (89)
Differentiate autoimmune hepatitis from primary biliary cholangitis	446 cases of WSI	The AUC of the external validation set was 0.81	Transformer	4 (Dx)	Gerussi et al. (90)
Preliminary MASLD image classification	six images representing histologically proven NAFLD	ChatGPT-4 achieved an ACC of 87.5%, while Google Bard achieved an ACC of 38.3%	Transformer, Self- Attention	4 (Dx)	Zhang et al. (91)
Predict HCC development in steatotic liver disease	Whole-slide images from 685 patients; retrospective multicenter pathology cohort; independent/unpaired validation cases	Training accuracy 81.0% and AUC 0.80; test accuracy 82.3% and AUC 0.84; future HCC development, patient-relevant prognostic endpoint; translational level 2, research model	DCNN	4 (Px)	Nakatsuka et al. (92)
WSI was used to predict activation of six immune gene features	4,888 cases of WSI	The AUC for predicting immune characteristic tumors was 0.91, and the AUC of the validation set was 0.92	CLAM	3 (Dx)	Zeng et al. (93)
Differentiate HCC from intrahepatic cholangiocarcinoma	785 WSI samples from 424 patients	Distinguish HCC from ICCA, the AUC reached 0.99	attention-MIL aggregation	4 (Dx)	Calderaro et al. (94)
Derive an atezolizumab-bevacizumab response biomarker	WSI samples from 424 patients	The mean Pearson correlation between the ABRS-*P* value and the ABRS score, the mean expression of ABRS genes, was r = 0.62	CLAM	3 (Px)	Zeng et al. (95)

CLAM, clustering-constrained attention multiple-instance learning; CNN, convolutional neural network; DCNN, deep convolutional neural network; Dx, diagnosis/monitoring question; GNN, graph neural network; H&E, hematoxylin and eosin; MIL, multiple-instance learning; OCEBM, Oxford Centre for Evidence-Based Medicine; Px, prognosis question; WSI, whole-slide image. For representative applications, study design, setting, validation, endpoint type, and evidence status are reported in the dataset and performance columns. Translational maturity levels are descriptive and defined in Section 1; no representative application demonstrated level 5 patient or health-system benefit. The OCEBM 2011 level was assigned by the primary clinical question and reported study design (28). Diagnostic-accuracy studies described as screening were appraised in the diagnosis/monitoring domain because the OCEBM screening hierarchy concerns whether early detection improves outcomes. Missing information on consecutive enrollment, blinding, and consistent reference-standard application was graded conservatively. OCEBM levels do not replace a study-specific risk-of-bias assessment.

**Table 3:** Summary and evidence appraisal of machine-learning models based on molecular biomarkers and structured clinical data in liver disease.

Focus	Dataset, design, and validation	Performance, endpoint, and translational status	Algorithm	OCEBM 2011 level	Reference
Predict the histological stage of fibrosis in patients with MASLD	1,370 patients with MASLD	AUC 0.86 for ≥ F2, 0.89 for ≥ F3, 0.89 for F4, and 0.80 for NAS ≥4 with fibrosis ≥ F2	RF	3 (Dx)	Chang et al. (104)
Predict the in-hospital mortality rate of patients with liver cirrhosis complicated with acute gastrointestinal bleeding	2,467 patients with AGIB cirrhosis	AUC = 0.986	LS-SVMR	2 (Px)	Bai et al. (105)
Predict in-hospital mortality in patients hospitalized with cirrhosis	7,239 inpatients at 115 centers; prospective global cohort; external validation in 28,670 US veterans	AUC 0.815 in CLEARED and 0.859 in the external cohort; in-hospital mortality, patient-relevant endpoint; translational level 3; no AI-assisted care-impact evaluation	Random forest	2 (Px)	Silvey et al. (114)
Risk assessment of HCC with low serum AFP levels in patients with hepatitis B virus-related cirrhosis	6,980 patients with hepatitis B virus-related cirrhosis	AUC = 0.832, sensitivity = 0.745, specificity=0.766	XGBoost	3 (Dx)	Xu et al. (106)
Early screening, diagnosis and recurrence monitoring of hepatocellular carcinoma in patients with chronic hepatitis B	397 patients with CHB-related liver cirrhosis and HCC	AUC = 0.967	RF	3 (Dx)	Su et al. (107)
Predict the risk of ACLF in WD patients.	208 patients with WD	AUC = 0.998, ACC=0.968	XGBoost	4 (Px)	Rao et al. (108)
Predict the prognosis of patients with ACLF after liver transplantation	1,384 patients with ACLF	One-year mortality AUC: EAML 0.707, RuleFit 0.719; 90-day mortality AUC: EAML 0.678, RuleFit 0.707	EAML; RuleFit	2 (Px)	Ge et al. (109)
Diagnosis and prognosis of HBV-ACLF	1,024 plasma samples from patients with acute exacerbation of HBV-related chronic liver disease	The AUC for diagnosing ACLF was 0.94, and the AUC for predicting 90-day mortality in ACLF patients was 0.87	RF	2 (Px)	Zhang et al. (110)
Predict the risk of recurrence after HCC surgery	231 patients	ACC of 82.2% and specificity of 85.7%	XGBoost	2 (Px)	Jia et al. (111)
Predict the liver-related prognosis after functional cure of chronic hepatitis B	4,787 consecutive patients with HBsAg seroclearance	c-index was 0.82, AUC was 0.86	Gradient Boosting	2 (Px)	Hur et al. (112)
Predict 28-day all-cause mortality in patients with alcoholic cirrhosis	2,134 patients with alcoholic cirrhosis	Validation AUC: random forest 0.843, XGBoost 0.842, support vector machine 0.866	Random forest; XGBoost; support vector machine	2 (Px)	Lei et al. (113)
Predict the prognosis of patients with hepatocellular carcinoma after radiofrequency ablation	1,778 treatment-naïve HCC patients	Harrel's c-index was 0.69	Transformer	2 (Px)	Sato et al. (115)
Predict the risk of HCC in patients with HCV-related cirrhosis	48,151 patients with HCV-related cirrhosis	AUC=0.759,Brier score=0.136	RNN	2 (Px)	Ioannou et al. (116)
Predict the risk of HCC after achieving SVR in individual patients	1,742 patients with chronic hepatitis C who achieved SVR	C-index = 0.839	RSF	2 (Px)	Minami et al. (117)

ACLF, acute-on-chronic liver failure; AGIB, acute gastrointestinal bleeding; Dx, diagnosis/monitoring question; EAML, expert-augmented machine learning; LS-SVMR, least-squares support vector machine regression; OCEBM, Oxford Centre for Evidence-Based Medicine; Px, prognosis question; RF, random forest; RSF, random survival forest; SVM, support vector machine; SVR, sustained virologic response; WD, Wilson disease; XGBoost, extreme gradient boosting. For representative applications, study design, setting, validation, endpoint type, and evidence status are reported in the dataset and performance columns. Translational maturity levels are descriptive and defined in Section 1; no representative application demonstrated level 5 patient or health-system benefit. The OCEBM 2011 level was assigned by the primary clinical question and reported study design (28). Diagnostic-accuracy studies described as screening were appraised in the diagnosis/monitoring domain because the OCEBM screening hierarchy concerns whether early detection improves outcomes. Missing information on consecutive enrollment, blinding, and consistent reference-standard application was graded conservatively. OCEBM levels do not replace a study-specific risk-of-bias assessment.

Each study in Tables 1–3 was also assigned a level using the 2011 Oxford Centre for Evidence-Based Medicine (OCEBM) framework according to its primary clinical question and reported study design (28). Diagnostic-accuracy studies described as screening were appraised in the diagnosis/monitoring domain because the OCEBM screening hierarchy addresses whether early detection improves outcomes. When consecutive enrollment, blinding, consistent application of a reference standard, or an inception cohort was not clearly reported, the OCEBM level was assigned conservatively. These design-based levels complement, but do not replace, a study-specific risk-of-bias assessment.

The representative evidence remains concentrated at translational maturity levels 1–3. Several multicenter studies include external or prospective testing, but few have evaluated whether AI-assisted care changes treatment, clinical outcomes, or resource use. Device authorization supports use for a defined indication, but it should not be interpreted as proof of patient benefit. When a study's design or validation status was not clear, its translational maturity was classified conservatively.

## 3 AI-based analysis of medical imaging and digital pathology in liver disease

Image-based assessment in hepatology includes ultrasound (US), CT, MRI, and histopathology. These fields are undergoing a data-driven, AI-enabled shift because they depend heavily on visual assessment and morphologic interpretation. Interpretation of radiologic images, ultrasonographic examinations, and histologic slides has traditionally depended on clinicians' experience and judgment, leading to interobserver variability and limitations in quantitative assessment (29–31). Computer vision converts images into machine-readable data and enables quantitative analysis that may improve the consistency of liver disease assessment (24, 32, 33). In this review, AI-based image analysis encompasses computer vision, radiomics, and computational pathology methods used to extract quantitative features from radiologic and histologic images. This section reviews applications in liver radiology, US, and digital pathology, ranging from feature extraction in radiologic images to large-scale analysis of whole-slide images. It also considers the scientific and system-level barriers to clinical integration.

### 3.1 AI-based analysis of noninvasive medical imaging

Noninvasive imaging includes US, CT, MRI, positron emission tomography (PET), elastography, and related techniques. These methods support HCC detection and characterization (34, 35), quantitative assessment of steatosis (36–38), and noninvasive assessment of fibrosis (39–41). Conventional interpretation depends on expert review and is affected by interoperator variability and limited quantitative reproducibility (29). AI-based image analysis can automatically extract information from digital images and generate quantitative features. Analysis of standardized Digital Imaging and Communications in Medicine (DICOM) data may produce reproducible, high-throughput imaging features that support diagnosis, risk prediction, and disease management (42, 43). Such tools may improve parts of the radiology workflow (44). Representative applications are summarized below.

#### 3.1.1 Ultrasound

Advances in algorithms and the availability of larger US datasets have supported the development of AI models for liver disease assessment (45). Model-DCB classified six types of focal liver lesions with accuracies of 0.85–0.86 across three external test cohorts in the reported multicenter study (46). A deep convolutional neural network developed for hepatic echinococcosis outperformed senior radiologists from a highly endemic area in differential diagnosis, classification, and lesion-activity identification (AUC, 0.942 vs. 0.844; *p* = 0.027). It also improved the AUC for junior radiologists from 0.743 to 0.850 and for attending radiologists from 0.808 to 0.886, suggesting possible utility in settings with limited specialist expertise (47). A sequential algorithm evaluated in 3,067 patients achieved 94% specificity and a 73% positive predictive value for all-cause advanced liver fibrosis while reducing unnecessary referrals by 35% (41). ModelURC, developed from grayscale US data, was evaluated for classifying HCC measuring 3 cm or less and may help radiologists with different levels of experience identify small HCCs (48). A related ML study assessed indeterminate hepatic nodules and supports further evaluation of AI-assisted US classification (49). AI methods for US assessment of MASLD have also been reviewed (50). Velacur One, evaluated against biopsy (51), received US FDA 510(k) clearance in 2025 to estimate liver stiffness, attenuation, and fat fraction as an aid used with other clinical indicators (52); authorization does not by itself demonstrate improved patient outcomes. Video-based AI for intraoperative liver US remains an emerging research area and requires further clinical validation (53, 54). EchoNet-Liver detected cirrhosis and steatotic liver disease from routine transthoracic echocardiograms containing subcostal liver views in a retrospective two-center study (55). Prospective impact studies and regulatory review would be required before routine deployment.

#### 3.1.2 MRI

AI-based MRI analysis may support the diagnosis and prognostic assessment of liver disease. In an independent external validation set containing data from multiple centers, scanners, and pathologic categories, an AI model achieved an AUC of 0.97, a sensitivity of 91.6%, and a specificity of 90.7% for malignant nodules. Model assistance increased radiologists' sensitivity from 72.3% to 74.0% to 85.7%–89.1% while maintaining specificity above 93%, suggesting potential value for identifying early HCC and atypical lesions (35). A deep learning-accelerated MRI protocol achieved sensitivity comparable to that of conventional protocols for malignant focal liver lesions, including HCC, while improving image quality and reducing scan time by approximately 50% (56). Separately, an explainable multiparametric MRI model achieved mean Dice coefficients of 0.98 and 0.96 for liver and tumor segmentation and classification accuracies of 93% and 97% for imaging features and lesions in external and prospective testing (57). A multicenter study also found that a deep learning model could categorize liver lesions on noncontrast MRI (58). These models may assist with differential diagnosis and surveillance for HCC and liver metastases, but further validation is required before they can be used independently in real-world settings. Standards for clinical licensing also remain to be established.

Che et al. (59) reviewed MRI-based AI studies for prognostic assessment in HCC. The CirrMRI600 database includes MRI data from patients with cirrhosis and 11 deep learning algorithms for segmentation (60), which may support future work on longitudinal monitoring. AI approaches have also been studied for fatty liver disease and hepatitis (61, 62). Deep learning applied to liver MRI has been used to predict cardiovascular risk (63). However, few studies have translated MRI-derived predictions into individualized treatment decisions. Prospective multicenter studies are needed before these models can be incorporated into routine imaging workflows as complementary tools for liver disease management (64).

#### 3.1.3 CT

CT-based AI models have been evaluated for HCC risk assessment, lesion classification, tumor segmentation, and, in some applications, reduction of radiation exposure. The DRCDS model diagnosed liver fibrosis more accurately than senior radiologists in the reported study (65). In the PLAN-B-DF study, the 10-year incidence of HCC was 46.2% in the high-risk group and 0.0% in the minimal-risk group; the low-risk group had an incidence of 0.4%. The model achieved a C-index of 0.91 in internal validation and 0.89 in independent external validation (66). These findings support further evaluation of CT-derived biomarkers for individualized HCC surveillance, although prospective evidence of clinical benefit is still required. For the differential diagnosis of focal liver lesions, LiAIDS achieved performance comparable to that of senior physicians, with an F1 score of 0.692 for malignant lesions, compared with 0.230–0.360 for junior radiologists (67). The system classified approximately 76% of patients as low risk during triage and achieved a negative predictive value of up to 99%, suggesting possible utility for clinical decision support and resource allocation (67). LiLNet achieved an accuracy of 94.7% and an AUC of 97.2% for differentiating benign from malignant liver tumors (68). The SALSA system, based on an improved U-Net architecture, achieved patient-level tumor-detection accuracy above 99% and a Dice similarity coefficient of 0.760 for tumor segmentation. It may provide quantitative information for preoperative planning, navigation, and treatment assessment (69). A multicenter CT-based deep learning algorithm for HCC diagnosis outperformed radiologic interpretation in internal validation and was noninferior in external testing (70). AI analysis of CT and other imaging modalities may also support diagnosis and outcome prediction in portal hypertension, for which current assessment may require invasive procedures (71). Future studies should prioritize prospective multicenter validation and assess automatic report generation, prediction of treatment response, and integration with multi-omics data.

#### 3.1.4 PET

In a clinical validation study of 120 patients, Long et al. (72) found that the RaDynPET deep learning model reduced PET acquisition time from 120 s to 30 s while maintaining agreement in standardized uptake value measurements and image quality. The findings suggest that the model may improve imaging efficiency and could permit shorter examinations or lower radiotracer doses without a marked loss of diagnostic quality. Such an approach may improve access to PET, shorten waiting times, and reduce workload in high-volume imaging departments. Further studies should assess generalizability across cancer types, scanners, and acquisition settings and evaluate integration with routine imaging workflows.

Together, these studies indicate that AI-based radiologic image analysis may improve analytical accuracy, support risk stratification, and streamline parts of the clinical workflow. However, the evidence is heterogeneous, and strong performance metrics alone do not establish patient-level benefit or readiness for routine clinical use.

Although many radiology AI models have reported strong performance in hepatology studies, clinical translation remains limited by data robustness, clinical validation, and integration with healthcare systems. Data robustness is a major constraint because model performance depends on the characteristics of the training data. Differences in scanner vendors, acquisition protocols, contrast administration, and reconstruction algorithms create domain shifts across institutions (73–75). These differences alter image texture and feature representation and can reduce the performance of models developed in single-center retrospective datasets when they are tested externally. Many tasks also require large, carefully annotated datasets. Pixel-level annotation for liver tumor segmentation or fibrosis staging is time-consuming, requires specialist expertise, and is affected by interobserver variability. Annotation noise and limited dataset size can therefore restrict scalability and real-world robustness (76, 77). In addition, many existing models target a single lesion type in one imaging modality, which limits their clinical scope.

The level of clinical evidence presents a separate limitation. Most published studies are retrospective proof-of-concept investigations that emphasize measures of diagnostic accuracy (78). Such studies are necessary during model development but do not demonstrate benefit to patients. Prospective multicenter studies, including randomized trials where appropriate, are needed to determine whether AI-assisted interpretation leads to earlier treatment, fewer diagnostic delays, improved survival, or more efficient use of healthcare resources. Evidence of clinical value beyond an incremental increase in analytical performance will be required for inclusion in clinical guidelines and reimbursement decisions. Future work should evaluate semi-supervised and related learning methods that reduce annotation requirements, conduct rigorous multicenter prospective validation, and develop tools that can be integrated into clinical workflows without compromising usability.

### 3.2 AI in digital pathology for liver disease

Histopathology is central to modern medicine and remains the reference standard for diagnosing many diseases. Pathologists have traditionally examined glass slides by microscopy, but this workflow is constrained by physical handling, limited accessibility, and workload (79). Diagnostic accuracy also depends on the pathologist's knowledge and experience (80), while demand for testing may exceed available specialist capacity. Digital pathology, centered on whole-slide imaging (WSI), converts glass slides into digital images (81, 82). This permits remote consultation, improves access, and, subject to regulatory approval, enables AI-assisted analysis (Table 2). In hepatology, histopathology is the reference standard for diagnosing and staging fibrosis, MASH, and HCC (5). Integrating computer vision and computational pathology with digital pathology may improve efficiency and reduce interobserver and intraobserver variability in conventional histopathologic assessment (83–86). As the volume of pathology data increases and specialist resources remain limited, these methods may support diagnosis, prognostic assessment, and therapeutic research in liver disease (87).

Computer vision models have been developed to support standardized assessment of liver fibrosis, MASH, and autoimmune liver diseases. The HOTSPoT model automatically segmented portal areas to support quantitative fibrosis assessment and achieved a Dice coefficient of 0.92 and an intersection-over-union score of 0.86 (88). AIM-MASH was designed to reduce interobserver variability in histologic assessment. For all features other than hepatocyte ballooning, for which the true-positive rate was 0.87, the reported classification measures for positive and negative examples exceeded 0.92 (89). The ALNE model used unsupervised learning to distinguish autoimmune hepatitis from primary biliary cholangitis and achieved an AUC of 0.81 in an external validation set, indicating some generalizability (90). In a small preliminary image-classification exercise, a general-purpose LLM achieved an accuracy of 87.5% on WSI from patients with MASLD; this proof-of-concept finding requires validation in substantially larger datasets (91). Although these models use different methods and address different tasks, they share the aim of reducing subjective variation through quantitative and automated analysis.

One HCC risk model maintained an accuracy of 82.3% and an AUC of 0.84 when evaluated on independent cases, suggesting some robustness across clinical settings but not yet establishing readiness for clinical use (92). The CLAM framework extended computational pathology to analysis of the immune microenvironment on routine histologic slides and achieved an AUC of 0.92 in independent validation (93). An attention-based multiple-instance learning model differentiated HCC from intrahepatic cholangiocarcinoma with an AUC of 0.99, and its predictions were associated with survival outcomes and spatial genomic features (94). These studies demonstrate research potential for risk prediction, immune profiling, and molecularly informed tumor classification. Their clinical actionability requires prospective validation.

The ABRS-*P* model was developed to derive pathologic biomarkers associated with response to targeted therapy (95), while the AIM-MASH continuous scoring system has been used to improve the standardization and objectivity of histologic assessment in drug trials (89, 96, 97). These approaches may support more reproducible assessment of treatment response, but their effect on treatment selection and patient outcomes remains to be established.

Although AI has advanced within digital pathology, several structural barriers limit clinical implementation. These include internal limitations in model development and validation and external barriers to workflow integration. During model development, technical heterogeneity in preprocessing creates domain shift (98). Differences in staining protocols, scanner models, and acquisition parameters alter the color distribution and texture of WSI and can reduce cross-center generalizability (99, 100). Conventional analysis of two-dimensional tissue sections also loses three-dimensional spatial information and may therefore miss morphologic features such as the continuity of vascular invasion (87, 101). High-resolution three-dimensional imaging methods, including light-sheet fluorescence microscopy, can partly address this limitation, but their cost and technical complexity restrict routine use and the creation of large, high-quality datasets. Annotation is another constraint. Pixel-level labeling of tissue morphology requires experienced pathologists and substantial time (102). Weakly supervised learning reduces the need for detailed annotation, but models trained in this way may overfit dataset-specific biases or background noise, resulting in poor performance during external validation (103). Finally, WSI files are large and require substantial storage, transmission capacity, and computational resources.

In summary, integrating AI-based computational pathology with digital pathology may reduce subjectivity and improve the reproducibility of histologic assessment. Automated quantitative analysis has shown potential for fibrosis staging, MASH assessment, and prediction of prognosis or treatment response in liver cancer. Progress will require robust cross-center methods, prospective multicenter validation, and tools designed around clinical workflows. These steps are necessary before technical performance can be translated into reliable benefits for patients.

## 4 AI-based models using molecular biomarkers and structured clinical data in liver disease

Unlike imaging data, molecular biomarker measurements and structured clinical variables are generally numerical and can be analyzed directly by ML models (Table 3). Omics technologies also produce high-dimensional datasets that are suitable for ML analysis, and these methods are beginning to influence liver disease research (104). In an international multicenter cohort of patients with cirrhosis and acute gastrointestinal bleeding, a least-squares support vector machine regression model stratified patients into low-, intermediate-, and high-risk groups and achieved an AUC of 0.986 (105). For noninvasive assessment of fibrosis in MASLD, a random forest model achieved AUCs of 0.86, 0.89, and 0.89 for fibrosis stages ≥ F2, ≥F3, and F4, respectively, and an AUC of 0.80 for the composite endpoint of a NAFLD activity score ≥4 with fibrosis ≥ F2 (104). An extreme gradient boosting (XGBoost) model developed for patients with HBV-related cirrhosis and low alpha-fetoprotein (AFP) levels achieved an AUC of 0.832 and a validation AUC of 0.829, compared with 0.658 for AFP alone (106). In another cohort of patients with HBV-related cirrhosis and low AFP levels, three serum N-glycomics-based models, HCC-GRF, HCC-GSVM, and prog-G, achieved AUCs of 0.967, 0.908, and 0.798, respectively, compared with 0.687 for AFP (107). These results support further evaluation of models that combine molecular markers and structured clinical data for earlier HCC detection, risk stratification, and prognosis.

Six ML models were evaluated for predicting acute-on-chronic liver failure (ACLF) in patients with Wilson disease, with XGBoost showing the best performance (AUC, 0.998; accuracy, 0.968) (108). Expert-augmented machine learning (EAML) was developed from data on 1,384 patients to predict survival after liver transplantation for ACLF (109). The EAML model achieved AUCs of 0.707 and 0.678 for 1-year and 90-day mortality, respectively, whereas the underlying RuleFit model achieved 0.719 and 0.707. Thus, expert augmentation did not improve discrimination over RuleFit in that cohort, although it may improve the clinical interpretability of extracted rules (109). Another study developed an ML model based on metabolomic markers that diagnosed pre-ACLF with an AUC of 0.940 and predicted 90-day mortality among patients with ACLF with an AUC of 0.870 (110).

ML models have also been evaluated for postoperative recurrence and other adverse outcomes. A tumor immune microenvironment spatial score, based on the spatial expression of biomarkers, predicted HCC recurrence with an accuracy of 82.2% and a specificity of 85.7% in a validation cohort of 231 patients drawn from five centers (111). A multinational study evaluated liver-related outcomes, including HCC, decompensation, and liver-related death, after functional cure of chronic hepatitis B (112). Its gradient boosting model achieved a C-index of 0.82 and an AUC of 0.86, compared with C-indices of 0.63–0.70 and AUCs of 0.62–0.72 for earlier HCC prediction models. The model may support individualized surveillance after HBsAg seroclearance (112). For short-term prognosis in alcohol-related cirrhosis, Lei et al. compared several models in MIMIC-IV data. In the validation set, the AUCs were 0.843 for random forest, 0.842 for XGBoost, and 0.866 for the support vector machine (113). Reporting validation rather than selected training performance provides a more conservative estimate of discrimination.

A recent prospective global study illustrates a higher level of prognostic evidence. The CLEARED cohort enrolled 7,239 inpatients with cirrhosis from 115 centers; a random forest model achieved an AUC of 0.815 for in-hospital mortality and was externally validated in 28,670 US veterans with an AUC of 0.859 (114). The patient-relevant endpoint, prospective enrollment, and external validation strengthen the evidence, although the predominantly male external cohort and absence of an AI-assisted care-impact study still limit claims about clinical benefit.

ML methods for longitudinal data may support dynamic risk assessment. A Transformer-based survival model generated individualized survival curves and dynamic risk estimates after radiofrequency ablation in a cohort of 1,778 patients with HCC. Its C-index was 0.69 in external validation, suggesting that it may help identify time windows for postoperative intervention (115). Deep learning models using longitudinal EHR data have also been evaluated for HCC prediction in patients with HCV-related cirrhosis and performed better than conventional regression models in the reported study (116). The SMART model predicted HCC after HCV eradication with a C-index of 0.839 and assessed recurrence risk in patients with early HCC with an AUC of 0.707 (117).

Numerical biomarker and multi-omics data have supported the development of many ML models. Because liver diseases change over time and vary by etiology and geographic region, future models should incorporate longitudinal data and undergo validation across regions and populations. Models intended to select individualized treatments or monitor treatment response will require prospective evaluation before clinical benefit can be established.

## 5 Large language models for hepatic electronic health record analysis

EHRs have evolved from digital versions of paper records into repositories of health information collected across the life course. In liver disease, EHRs contain structured variables and unstructured text, including progress notes, discharge summaries, pathology reports, and endoscopy reports. These sources record information such as hepatic encephalopathy grade, ascites severity, and esophageal variceal morphology, all of which may affect prognosis. The scale and complexity of EHR data make them difficult to use. Data are heterogeneous and fragmented, recording standards vary among healthcare settings, and information is often distributed across separate systems. Much clinically relevant information is embedded in free text, and manual extraction is inefficient and difficult to scale. Missing data, delayed diagnosis, selection bias, and sex-related bias can also affect model performance. These problems have been reported in EHR-based models for predicting HCC in patients with MASLD (118). Teles et al. (119) reviewed multimodal deep learning methods and the challenges of EHR data fusion.

Transformer-based LLMs, including ChatGPT, Gemini, and DeepSeek, can generate text, synthesize information, and answer questions. LLMs are therefore a subclass of Transformer models rather than a parallel model family. Their hepatology applications should be evaluated according to the task performed, the source and sensitivity of the input data, the intended user, and the consequences of error. The following task-based organization distinguishes patient-facing communication, retrieval-augmented guideline support, EHR phenotyping and information extraction, and report classification or multimodal decision support.

### 5.1 Patient-facing chatbots and health information

Patients increasingly use general-purpose LLMs to obtain medical information (120). These models are trained on broad corpora and may lack the depth, currency, or rigor required for specialist decisions (121–125). They can produce inaccurate, outdated, or fabricated information in plausible language, particularly for complex or uncommon liver diseases (126, 127), and may amplify convincing misinformation (128). Evaluation should therefore measure factual accuracy, uncertainty communication, readability, harmful advice, and escalation to qualified clinicians. Patient-facing systems should not be presented as autonomous diagnostic or treatment tools.

### 5.2 Retrieval-augmented guideline support

Retrieval-augmented generation (RAG) supplies an LLM with selected external documents at the time of a query. Ge et al. developed LiVersa, a liver disease-specific RAG interface, and hepatologists judged its answers more accurate but less comprehensive and safe than some general-purpose outputs; the authors described it as a proof of concept requiring further refinement (129). Kresevic et al. combined GPT-4 Turbo with RAG and prompt engineering to interpret chronic HCV guidelines (130). Such systems require evaluation of source coverage, retrieval accuracy, citation fidelity, version control, and performance after guideline updates before live decision support.

### 5.3 EHR phenotyping and information extraction

NLP and LLM systems can convert unstructured EHR text into structured variables, identify clinical phenotypes, and reduce manual chart review. LLMs have been evaluated for extracting social determinants of health from clinical notes across institutions (131), while a GPT-4 approach identified and classified cirrhosis cases from EHR data (132). Earlier NLP and more recent EHR algorithms have also been studied for identifying and monitoring MASLD (133, 134). Validation should report document sampling, annotation standards, positive predictive value, missed cases, portability across institutions, and privacy safeguards; high note-level accuracy does not ensure unbiased cohort identification.

### 5.4 Report classification and multimodal decision support

Spitzl et al. used LLMs to classify liver lesions from MRI reports (135), a task in which the input is a radiologist's text and the output is a diagnostic category rather than direct image interpretation. Multimodal systems may additionally combine reports, images, laboratory data, and longitudinal records. These tasks carry different error pathways and should be validated separately for calibration, subgroup performance, robustness to missing inputs, and the effect of human review. No current evidence supports replacing specialist interpretation with an autonomous LLM.

## 6 Translational prospects

Figure 1 summarizes potential AI applications across the continuum of liver disease management and explicitly distinguishes four stages: documented clinical use or regulatory authorization, prospective or registered clinical evaluation, retrospective proof-of-concept, and exploratory or conceptual directions. Maturity is assigned from the cited evidence and intended use rather than from predictive performance alone; it may differ by jurisdiction, institution, and product version.

**Figure 1:**
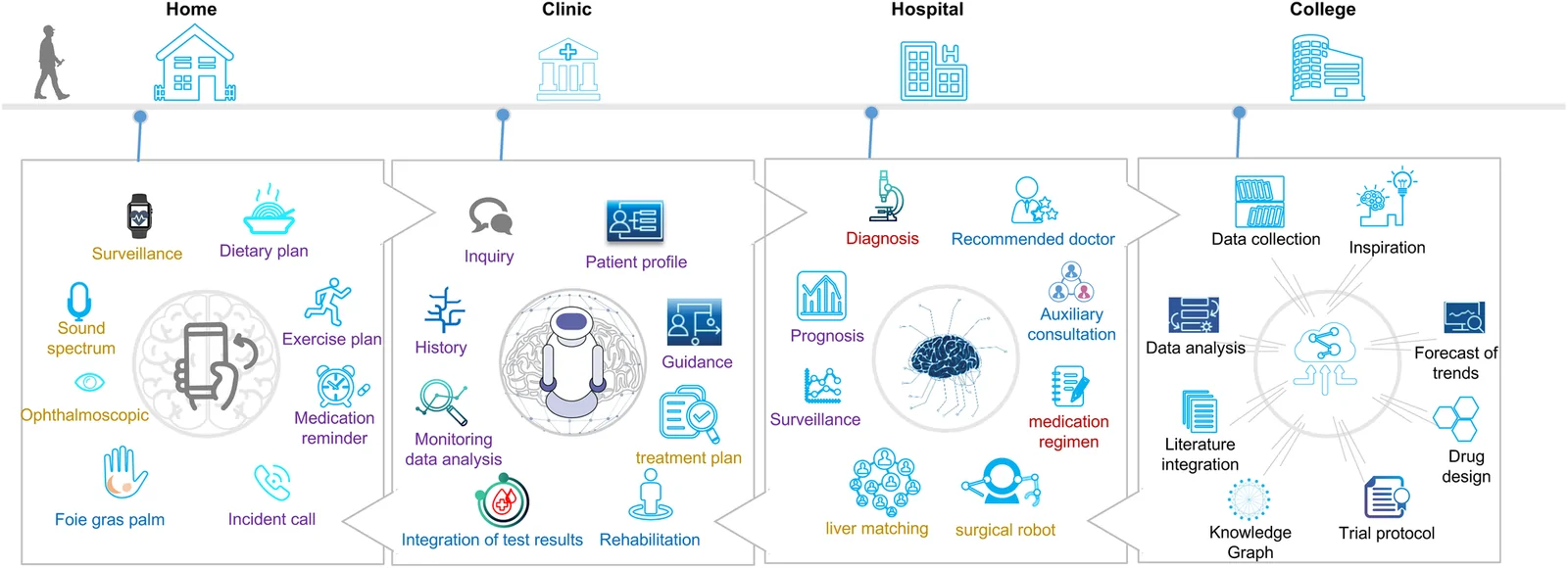
Potential application settings and evidence maturity for artificial intelligence in hepatology. Visual coding distinguishes documented clinical use or regulatory authorization, prospective or registered clinical evaluation, retrospective proof-of-concept, and exploratory or conceptual directions. Regulatory status, validation, and intended use may differ by jurisdiction, institution, and product version. The four colors of the character represent different levels of clinical maturity: dark red indicates that it has been clinically used; Brown represents entry into the prospective evaluation or registered clinical trial phase; Purple represents retrospective proof-of-concept application; Blue represents exploratory or conceptual applications.

**Figure 2:**
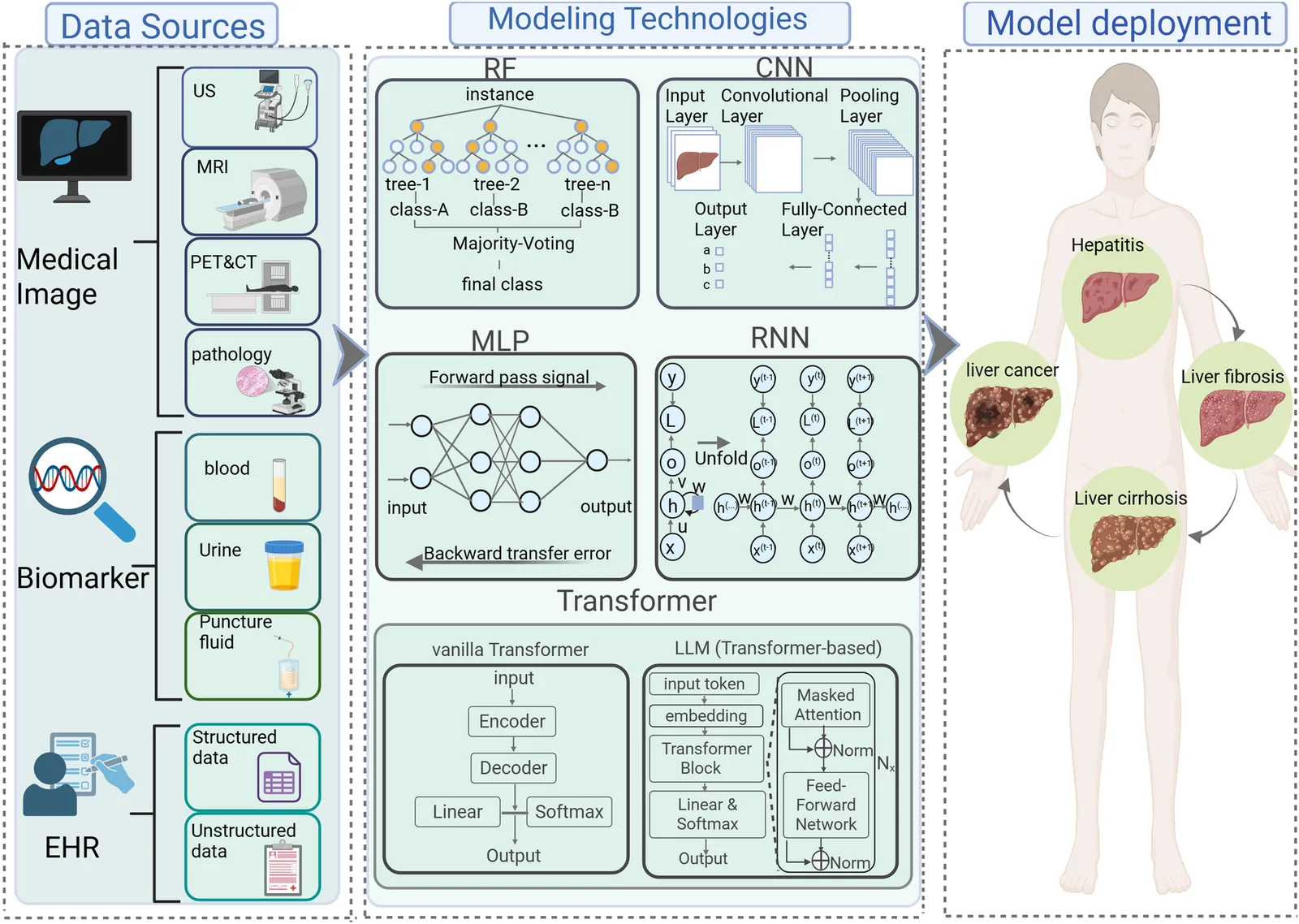
Data sources, model families, and clinical tasks for AI in liver disease. The figure is organized as data source to model family to clinical task or application. Data sources include radiologic images, digital pathology, molecular and omics measurements, and EHR or longitudinal data. Model families are shown hierarchically: LLMs are nested under Transformer-based models, and retrieval-augmented generation is shown as an external evidence-retrieval layer. Clinical tasks include detection, classification, segmentation, quantification, prognosis, information extraction, guideline support, treatment planning, and workflow support.

### 6.1 Documented clinical use or regulatory authorization

Documented liver-specific authorization remains limited. Velacur One is a prescription ultrasound device cleared by the US FDA to estimate liver tissue stiffness, attenuation, and fat fraction as an aid used with other clinical indicators (51, 52). Selected imaging and decision-support tools have also entered local hepatology workflows (136), but local adoption does not establish generalizability or patient benefit. No currently cited system is authorized to make an independent diagnosis or treatment decision without professional interpretation. Deployment should therefore remain within the defined indication, operator requirements, and institutional governance process.

### 6.2 Prospective evaluation and registered clinical studies

Several nontraditional screening approaches have reached human or prospective evaluation but remain investigational. These include wearable or on-skin sensors for physiologic monitoring (137), tongue-image models for liver fibrosis or MASLD (138, 139), ocular-image screening for hepatobiliary disease (140), speech analysis for hepatic encephalopathy (141, 142), and sclera or urine-image analysis for jaundice (143). A home-monitoring app is also being studied in a registered trial (NCT06920316). Future studies should compare these tools with current care pathways and measure false reassurance, unnecessary referral, adherence, clinical actions, and patient outcomes rather than accuracy alone.

Prospective evaluation is also needed for hospital decision support. Models have been developed to compare resection with transplantation in HCC (144), predict decompensation or liver outcomes (145, 146), reduce futile donor procurement and support donor-recipient matching (147, 148), map treatment regions (54), and assist real-time procedural imaging (149). These applications could affect allocation or invasive treatment and therefore require stronger calibration, safety, and impact evidence than low-risk administrative tools.

### 6.3 Retrospective proof-of-concept applications

Many home and primary-care concepts are supported mainly by retrospective, simulated, or experimental evidence. AI-generated dietary, sleep (150, 151), and exercise plans (152–156), nutrition guidance (157, 158), wearable-EHR alerts evaluated with simulated sensor data (159), patient education (160), and conversational diagnostic systems (161) may support care, but their effects on adherence, access, and liver-related outcomes remain uncertain. Proposed primary-care systems may assist patient navigation or clinician workload (162, 163), yet few have been evaluated in routine ambulatory hepatology.

Proof-of-concept work also includes multimodal biomarker discovery and data fusion (164–166), therapeutic-reasoning agents (167), and the MAGIC multi-agent system, which reported 91.6% diagnostic accuracy for complex liver diseases (168). Multimodal analysis may identify candidate therapeutic targets (169), and internet-enabled TCM systems have been evaluated for information or treatment-related tasks (170, 171). None of these reports establishes that an autonomous model can safely generate individualized treatment plans.

### 6.4 Exploratory or conceptual directions

Traditional observation, listening, questioning, and pulse assessment have been proposed as sources of model features (172–174). Cutaneous signs such as palmar erythema and spider angiomas (175), fetor hepaticus (176), and general olfactory representation learning (177) provide hypotheses for future screening, but odor-based or skin-sign diagnosis has not been clinically validated. Robotic surgery is clinician controlled (178, 179); autonomous replacement of surgeons remains conceptual and is not supported by current evidence. Rehabilitation reminders and monitoring after discharge may be useful, but escalation and accountability must remain explicit (180).

AI may also generate personalized lifestyle suggestions, combine continuous home data, or act as a virtual health coach. These ideas should be described as health-promotion or research concepts unless they have a defined medical indication, validated alert pathway, privacy protection, and evidence of benefit. Consumer availability should not be equated with clinical validation.

In research, AI can support literature mining and hypothesis generation (181), integration of multimodal biomedical and genomic data (182, 183), knowledge-graph construction and causal inference (184, 185), drug design and therapeutic reasoning (186–189), and virtual-liver, organoid, or organ-on-chip studies (190–192). AI has also been applied to clinical-trial design, participant recruitment, endpoint assessment, and safety monitoring (97, 193). These tools may improve research efficiency, but investigators retain responsibility for validity, protocol adherence, and interpretation.

AI-generated synthetic cohorts may partly address shortages of research data, support multi-institutional collaboration, and accelerate hepatology research (194). Synthetic data should be evaluated for fidelity, privacy leakage, preservation of clinically important rare events, and whether analyses reproduce conclusions from real patient data.

## 7 Challenges

Selected AI applications have begun to move from laboratory validation toward real-world deployment. Broader use remains limited by data heterogeneity, uncertain generalizability, limited trust among healthcare professionals, and regulatory frameworks that have not kept pace with the technology.

### 7.1 Data quality and availability

High-quality annotated data remain a central constraint on AI performance. Differences in equipment, scanning parameters, and preprocessing among hospitals create substantial data heterogeneity and can reduce model generalizability. For example, echo-intensity distributions differ systematically between GE and Siemens US systems at the same stage of fibrosis (195, 196). Future work should establish shared liver disease databases, define consistent standards for data collection, storage, and annotation, and promote interoperability standards such as DICOM and Health Level Seven Fast Healthcare Interoperability Resources (HL7 FHIR) (197, 198).

### 7.2 Model generalizability and robustness

AI models trained in one population or institution may not generalize to others because of demographic, ethnic, and technical differences. Many models perform well in single-center datasets but show lower performance during external validation (199). Domain adaptation, adaptive aggregation methods such as AdaFed, and local fine-tuning may improve consistency across institutions (200, 201).

### 7.3 Clinical integration and workflow compatibility

Clinicians may resist AI tools because of limited trust, poor usability, or disruption of established workflows (202). Providing SHAP values, heat maps, or counterfactual explanations may improve transparency. Interfaces compatible with HL7 FHIR could facilitate integration with existing hospital information systems and picture archiving and communication systems (203).

Overreliance by physicians is another potential risk (204). In smaller hospitals, AI assistance may produce modest improvements in work quality while reducing efficiency among less experienced physicians (205). Clinical implementation should therefore discourage uncritical reliance on automated recommendations and include appropriate physician training. Training should cover efficient system operation, interpretation of AI output, integration of recommendations into diagnostic reasoning, and the need to retain independent clinical judgment. AI should be used as a decision-support tool rather than as a substitute for clinical responsibility.

### 7.4 Regulatory, lifecycle, and accountability concerns

Regulation must address both initial authorization and changes made after release. Conventional medical-device pathways may not readily accommodate AI systems that are periodically updated or continuously learning (206, 207). The US FDA's final guidance on predetermined change control plans allows a manufacturer to specify planned modifications, the methods used to develop and validate them, and an impact assessment within a marketing submission (208). The International Medical Device Regulators Forum's good machine learning practice principles emphasize representative data, independent test sets, human-AI team performance, and monitoring of deployed models (209). WHO regulatory considerations likewise frame risk-benefit assessment and performance monitoring across diverse clinical and geographic settings (210).

Post-deployment validation should monitor three distinct dimensions: system integrity, predictive performance, and clinical impact (211). A monitoring plan should predefine input-data shifts, missingness, calibration, discrimination, subgroup performance, alert burden, override rates, downstream actions, and patient or workflow outcomes. It should specify measurement cadence, responsible personnel, escalation thresholds, and actions such as investigation, recalibration, rollback, or decommissioning. Model version, data provenance, local validation, and every material update should be documented; silent model changes should not be permitted in clinical use.

Responsibility for AI-assisted decisions is shared but should not be ambiguous. Manufacturers are responsible for the intended use, training data documentation, version control, and validated update procedures. Health systems are responsible for local integration, access control, cybersecurity, staff training, monitoring, incident response, and reporting. Clinicians remain accountable for interpreting the output in context and for overriding unsafe recommendations. Audit logs should preserve the model version, inputs, output, user action, and outcome. Generative systems require additional controls for hallucination, prompt injection, leakage of protected health information, and changes in upstream knowledge sources.

### 7.5 Risks to health equity

Most training data are obtained from large urban hospitals, while rural and primary-care populations remain underrepresented. This imbalance may introduce systematic bia (212). Dedicated funding for real-world studies in rural and primary-care settings may improve representativeness and support more equitable generalizability.

## 8 Conclusion

AI is changing the management of liver disease across diagnosis, risk assessment, treatment planning, and follow-up. Progress from research validation to broader clinical deployment will depend on evidence from external and prospective studies, integration with clinical workflows, and effective post-deployment oversight. Federated learning may reduce some barriers to data sharing, explainable AI may support clinician trust, and generative AI may enable new forms of patient support, but none of these approaches removes the need for clinical validation and human accountability. Clinicians should participate in the design and evaluation of AI systems and define relevant clinical requirements. Developers should understand clinical reasoning and prioritize interpretability and usability. Policymakers should develop adaptive regulatory pathways and address data sharing and reimbursement. Collaboration among these groups is necessary to build AI-supported hepatology systems that are safe, equitable, clinically useful, and centered on patient needs.
